# Efficacy of paclitaxel‐carboplatin with bevacizumab as a late‐line therapy for patients with advanced nonsquamous non‐small cell lung cancer: A platinum rechallenge

**DOI:** 10.1111/1759-7714.15107

**Published:** 2023-09-12

**Authors:** Jun Sugisaka, Yukihiro Toi, Yosuke Kawashima, Yutaka Domeki, Tomoiki Aiba, Sachiko Kawana, Atsushi Nakamura, Shinsuke Yamanda, Yuichiro Kimura, Shunichi Sugawara

**Affiliations:** ^1^ Department of Pulmonary Medicine Sendai Kousei Hospital Sendai Japan

**Keywords:** platinum, bevacizumab, paclitaxel, nonsmall cell lung cancer, cancer

## Abstract

**Background:**

There is no well‐established late‐line treatment for advanced nonsquamous non‐small cell lung cancer (NSCLC). Therefore, we retrospectively determined the efficacy and safety of platinum rechallenge with paclitaxel‐carboplatin and bevacizumab in patients with nonsquamous NSCLC as a late‐line therapy in a clinical setting.

**Methods:**

Thirty patients with nonsquamous NSCLC who received paclitaxel‐carboplatin with bevacizumab therapy as a late‐line treatment at Sendai Kousei Hospital (Miyagi, Japan) between December 2011 and December 2021 were enrolled into the study. The efficacy and safety of this treatment were evaluated. The patients were further categorized into responders and nonresponders, and predictive factors of treatment response were estimated.

**Results:**

The median progression‐free survival (PFS) was 6.3 (range, 4.9–6.8) months, and the median overall survival (OS) was 11.8 (range, 7.2–17.2) months. There were no significant differences in PFS and OS between patients with and those without epidermal growth factor receptor mutations. In the univariate analyses of this study, responders were younger than nonresponders (*p* = 0.012). No fatal adverse events were reported.

**Conclusions:**

With the increase in the number of treatment options in recent years, the sequence of treatments and overall therapeutic strategy are becoming increasingly important. Thus, platinum rechallenge with paclitaxel‐carboplatin and bevacizumab, a late‐line treatment for patients with nonsquamous NSCLC, may be an effective therapeutic option.

## Graphical abstract

- Thirty patients with nonsquamous non‐small cell lung cancer who received paclitaxel‐carboplatin with bevacizumab therapy as a late‐line treatment were retrospectively evaluated.
- The median progression‐free survival was 6.3 months.
- Paclitaxel‐carboplatin with bevacizumab as a late‐line therapy may be an effective therapeutic option.

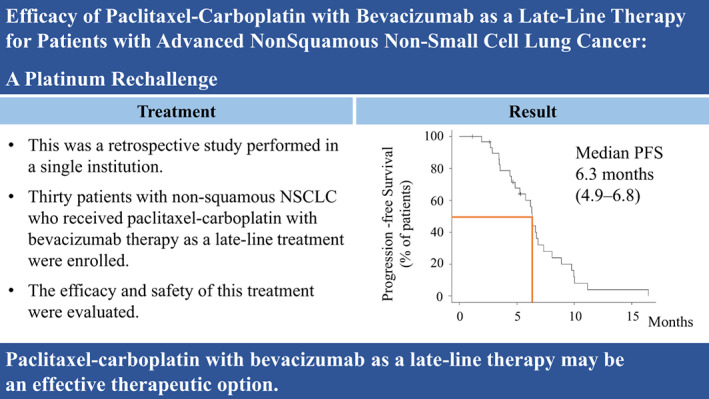

## INTRODUCTION

Lung cancer remains a leading cause of cancer‐related deaths worldwide.^1^ More than 80% of all lung cancers are non‐small cell lung cancers (NSCLCs). In addition, NSCLC can be broadly classified into two types, namely, squamous cell carcinoma and nonsquamous cell carcinoma. Several different therapies have been established for NSCLC, including chemotherapy, molecular targeted drugs, and immune checkpoint inhibitors (ICIs).^2^, ^3^

Above all, ICIs have become standard treatment options for NSCLC. In recent years, the life expectancy of patients with NSCLC has increased.^2^, ^3^ Based on the results of global clinical trials, such as KEYNOTE‐189,^4^ KEYNOTE‐407,^5^ IMpower130,^6^ and IMpower150,^7^ ICI plus chemotherapy has become the standard first‐line therapy for these patients. Moreover, these treatments have improved the prognosis of patients with NSCLC. Because more treatment options are now available, it is important to consider therapies that are most appropriate as late‐line therapy (the treatment after the second‐line therapy). However, there are considerable problems associated with conducting clinical trials of late‐line therapies in patients with advanced lung cancer. In particular, the matching of patient conditions is problematic.

Paclitaxel‐carboplatin with bevacizumab as the first‐line therapy for patients with nonsquamous NSCLC has significant survival benefits.^8^ Nonetheless, the use of this regimen has decreased in recent years because of the increased use of pemetrexed combination therapy as the first‐line treatment for advanced NSCLC. In addition, because of concerns about adverse events (AEs) associated with paclitaxel therapy, such as nervous system disorders, this regimen tends to be avoided in first‐line treatment. Here, we describe the clinical effects of paclitaxel‐carboplatin with bevacizumab as a late‐line therapy for patients with nonsquamous NSCLC. The aim of this study was to retrospectively determine the efficacy and safety of a platinum rechallenge with paclitaxel‐carboplatin and bevacizumab in patients with nonsquamous NSCLC.

## METHODS

This was a retrospective study of patients with nonsquamous NSCLC treated with paclitaxel‐carboplatin and bevacizumab as a late‐line therapy at Sendai Kousei Hospital (Miyagi, Japan) between December 2011 and December 2021. The data cutoff time was December 31, 2021. Patients were selected based on the following criteria: (1) pathologically proven stage IIIB–IV nonsquamous NSCLC, based on the tumor‐node‐metastasis (TNM) staging system (seventh edition),^9^ which includes postoperative recurrence, and (2) treatment with paclitaxel‐carboplatin with bevacizumab as a late‐line therapy. Patients received paclitaxel (200 mg/m^2^ body surface area) and carboplatin (area under the concentration–time curve, 6 mg/mL/min) plus bevacizumab (15 mg/kg) intravenously every 3 weeks for 4–6 cycles, followed by bevacizumab monotherapy every 3 weeks, with reference to the ECOG 4599 trial.^8^ This treatment was continued until disease progression or intolerable AEs occurred. The following data were collected from patient records: baseline characteristics, best response according to Response Evaluation Criteria in Solid Tumors (RECIST) version 1.1,^10^ and AEs according to the Common Terminology Criteria for Adverse Events (CTCAE) version 4.0. Using these data, we evaluated the objective response rate (ORR), disease control rate (DCR), progression‐free survival (PFS), overall survival (OS), and safety. The patients were further classified as responders who achieved complete response (CR) or partial response (PR), and nonresponders who developed stable disease (SD) or progressive disease (PD). Predictive factors of treatment response were also analyzed using univariate analyses.

This study was conducted according to the Helsinki Declaration and the study protocol was approved by the institutional review board of Sendai Kousei Hospital (IRB no.2‐37). Informed consent was obtained using the opt‐out method on the website of our institution. Those who opted out were excluded.

### Assessment

Objective tumor response to chemotherapy was assessed by computed tomography at 8–9‐week intervals. Two pulmonary physicians (an attending physician and an investigator) evaluated objective tumor response using the RECIST guidelines, version 1.1.^10^ The attending physician conducted a physical examination, assessed patients for AEs (at 3‐week intervals throughout the treatment course), and recorded the results.

### Statistical analysis

All statistical analyses were performed with EZR (Saitama Medical Center, Jichi Medical University, Saitama, Japan), a graphical user interface for R (R Foundation for Statistical Computing).^11^ Differences with *p* < 0.05, based on two‐tailed tests, were considered statistically significant. We estimated the time to events using the Kaplan‐Meier method. The hazard ratio was calculated using the Cox proportional hazards model.

## RESULTS

### Patient characteristics

Thirty patients with nonsquamous NSCLC treated with paclitaxel‐carboplatin and bevacizumab at Sendai Kousei Hospital (Miyagi, Japan) between December 2011 and December 2021 were enrolled in this study. Patient characteristics and comparisons between patients are summarized in Table 1. The median age was 66 (range, 51–79) years, and the study involved 22 men and eight women. The median number of prior regimens was 3.5 (range, 2–8). A total of 14 patients (46.7%) had epidermal growth factor receptor (EGFR)‐mutant tumors.

**TABLE 1:** Baseline characteristics of the study population (n = 30).

Characteristic	Value^a^
Median age, years	66 (51–79)
Sex (male), n (%)	22 (73.3%)
ECOG PS^b^	
0	13 (43.3%)
1	17 (56.7%)
2	0 (0%)
Smoking status	
Current smoker or ever smoked	20 (66.7%)
Never smoked	10 (33.3%)
Pathological subtype	
Adenocarcinoma	30 (100%)
EGFR status	
Mutant	14 (46.7%)
Clinical stage	
IIIA–IIIB	4 (13.3%)
IV	20 (66.7%)
Recurrence	6 (20.0%)
Number of prior regimens	
2	6 (20.0%)
3	9 (30.0%)
≥4	15 (50.0%)
Prior platinum formulation	
Administered	29 (96.7%)
None	1 (3.3%)
Prior docetaxel	
Administered	25 (83.3%)
None	5 (16.7%)
Prior EGFR TKI	
Administered	16 (53.3%)
None	14 (46.7%)
Prior ICI	
Administered	19 (63.3%)
None	11 (36.7%)

Abbreviations: ECOG, Eastern Cooperative Oncology Group; EGFR, epidermal growth factor receptor; ICI, immune checkpoint inhibitor; n, number; NSCLC, non‐small cell lung cancer; PS, performance‐status; TKI, tyrosine kinase inhibitor.

^a^ Values in the table are presented as median with the range in square brackets or as a number with the percentage in parentheses.

^b^ Eastern Cooperative Oncology Group performance status (ECOG PS) scores range from 0 to 4, with high numbers indicating high disability.

### Clinical outcome

**FIGURE 1:**
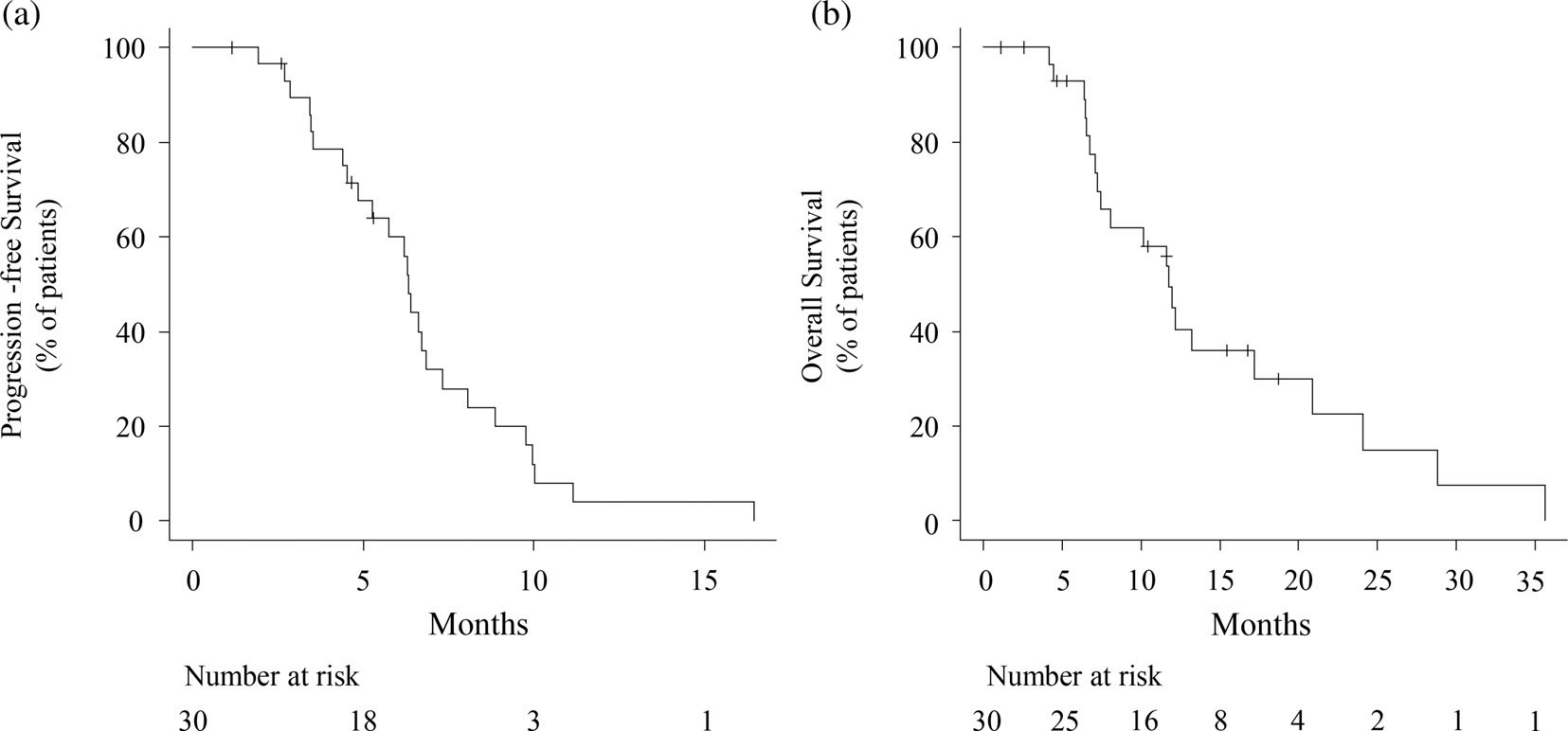
Survival in nonsquamous non‐small cell lung cancer (NSCLC) patients. (a) Progression‐free survival in nonsquamous NSCLC patients. (b) Overall survival in nonsquamous NSCLC patients. Ticks indicate patients whose data were censored on December 31, 2021.

**FIGURE 2:**
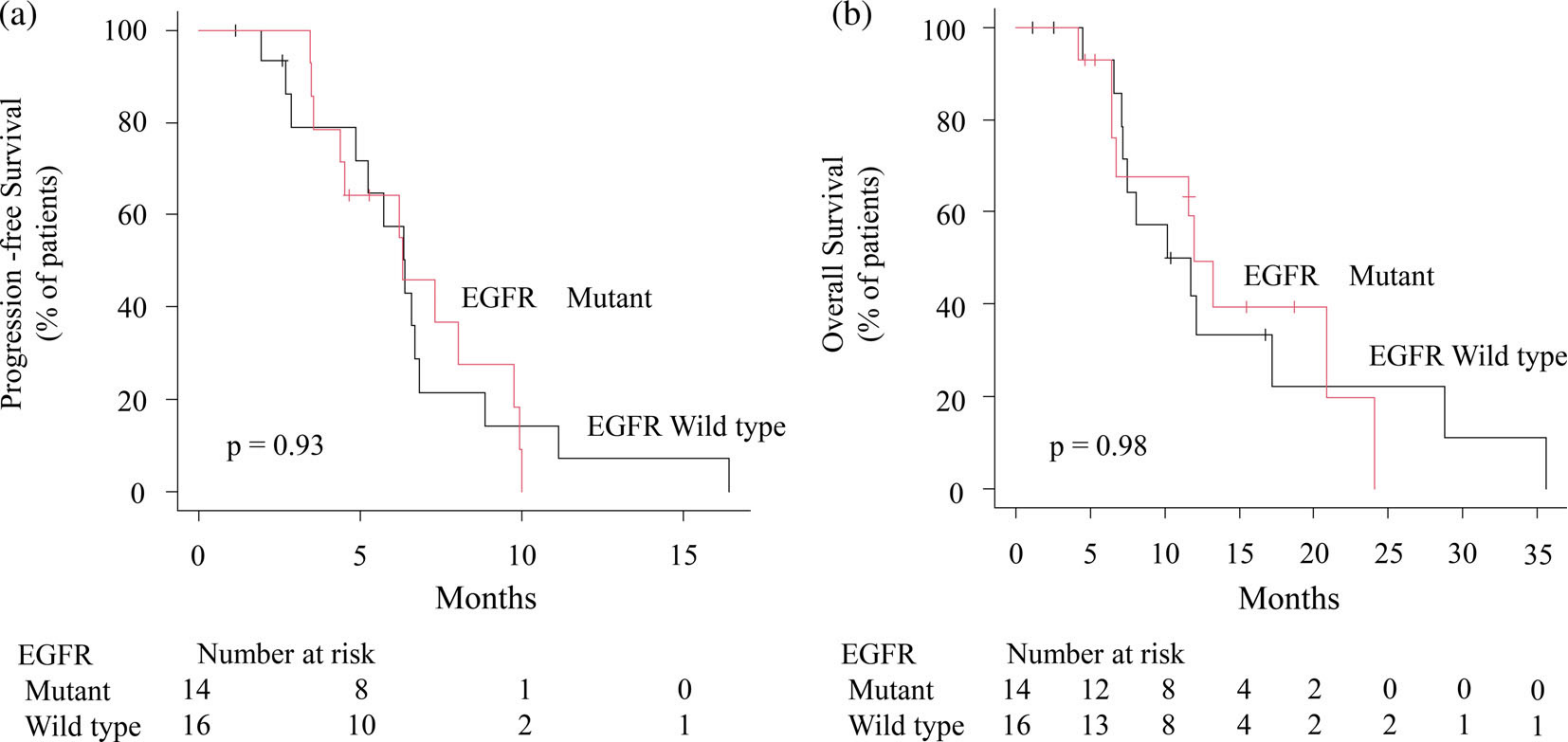
Survival in nonsquamous non‐small cell lung cancer (NSCLC) patients with or without the EGFR mutation. (a) Progression‐free survival in nonsquamous NSCLC patients with or without the EGFR mutation. (b) Overall survival in nonsquamous NSCLC patients with or without the EGFR mutation. Ticks indicate patients whose data were censored on December 31, 2021.

At data cutoff, the median follow‐up was 11.2 (range, 1.3–38.7) months. Overall, 26 patients (86.7%) were treated with four or more courses of chemotherapy. Four patients were still undergoing treatment. The median PFS was 6.3 (range, 4.9–6.8) months and the median OS was 11.8 (range, 7.2–17.2 months). The Kaplan Meier curves for PFS and OS are shown in Figure 1. Regarding the best treatment effect, although 20 (66.7%) of 30 patients achieved partial response, seven (23.3%) achieved stable disease as their best response. Three patients (10.0%) experienced disease progression without any effect of this therapy. None of the patients showed a complete response. The ORR was 66.7%, and the DCR was 90.0%. The Kaplan‐Meier curves of PFS and OS for patients with and without an EGFR mutation are shown in Figure 2. The median PFS was 6.3 and 6.4 months in patients with and without EGFR mutation, respectively. The median OS was 12.0 and 11.0 months in patients with and without EGFR mutation, respectively. No significant differences were observed in the PFS and OS between the groups (*p* = 0.93 and 0.98, respectively).

AEs of any grade were observed in 30 patients (100%) and severe AEs (grade ≥3) were observed in 21 patients (70.0%). The AE categories are shown in Table 2. Regarding the severe AEs (grade ≥3), 14 patients (46.7%) presented with neutropenia and seven (23.3%) demonstrated a decrease in white blood cells. Two patients (6.7%) developed febrile neutropenia. Twelve patients (40.0%) experienced grade 2 or higher peripheral sensory neuropathy. Epistaxis was reported in four patients (13.3%), but all cases were mild or moderate. Three patients (10.0%) experienced hypertension, and three patient (10.0%) presented with proteinuria. No fatal AEs were reported.

**TABLE 2:** Adverse events reported during the treatment of this study (n = 30).

	n (%)	Grade of AEs 1/2/3/4/5	Grade ≥3 n (%)
Peripheral sensory neuropathy	19 (63.3)	7/9/3/0/0	3 (10.0)
Neutropenia	15 (50.0)	0/1/6/8/0	14 (46.7)
Decreased white blood cell count	11 (36.7)	2/2/6/1/0	7 (23.3)
Anemia	11 (36.7)	5/4/2/0/0	2 (6.7)
Thrombocytopenia	8 (26.7)	6/1/1/0/0	1 (3.3)
Alopecia	6 (20.0)	5/1/0/0/0	0
Nausea	5 (16.7)	3/0/2/0/0	2 (6.7)
Anorexia	5 (16.7)	1/3/1/0/0	1 (3.3)
Malaise	4 (13.3)	2/2/0/0/0	0
Epistaxis	4 (13.3)	3/1/0/0/0	0
Hypertension	3 (10.0)	0/2/1/0/0	1 (3.3)
Proteinuria	3 (10.0)	3/0/0/0/0	0
Febrile neutropenia	2 (6.7)	0/0/2/0/0	2 (6.7)
Mucositis oral	2 (6.7)	0/2/0/0/0	0
Fatigue	2 (6.7)	1/1/0/0/0	0
Dizziness	2 (6.7)	1/1/0/0/0	0
Pain	2 (6.7)	1/1/0/0/0	0
Increased alkaline phosphatase level	2 (6.7)	2/0/0/0/0	0
Skin reaction	1 (3.3)	0/0/0/1/0	1 (3.3)
Aspartate aminotransferase increased	1 (3.3)	1/0/0/0/0	0
Allergic reaction	1 (3.3)	0/1/0/0/0	0
Lung infection	1 (3.3)	0/1/0/0/0	0
Myalgia	1 (3.3)	1/0/0/0/0	0

Abbreviation: n, number.

Table 3 shows a comparison of patient characteristics between responders and nonresponders. No significant difference was observed in terms of sex, ECOG PS, smoking history, EGFR status, clinical stage, the number of prior regimens, or prior regimen between the two groups. Univariate analysis identified that age was a significant difference between responders and nonresponders (*p* = 0.012).

**TABLE 3:** Characteristics of responders and nonresponders (n = 30).

Characteristic, n (%)	Responder n = 20	Nonresponder n = 10	*p* value
Median age, years	61.5 (51–69)	67 (56–79)	0.012^c^
Sex (male)	15 (75.0%)	7 (70.0%)	1^b^
ECOG PS^a^, 0	11 (50.5%)	2 (20.0%)	0.119^b^
Smoking status (ex, current)	12 (60.0%)	8 (80.0%)	0.419^b^
EGFR status, mutant	11 (50.5%)	3 (30.0%)	0.26^b^
Clinical stage			1^b^
IIIA–IIIB	3 (15.0%)	1 (10.0%)	
IV	13 (65.0%)	7 (70.0%)	
Recurrence	4 (20.0%)	2 (20.0%)	
Number of prior regimens, median	3.5 (2–8)	3.5 (2–5)	0.875^c^
Prior docetaxel	15 (75.0%)	10 (100%)	0.14^b^
Prior EGFR TKI	11 (55.0%)	5 (50.0%)	1^b^
Prior ICI	13 (65.0%)	6 (60.0%)	1^b^

Abbreviations: ECOG, Eastern Cooperative Oncology Group; EGFR, epidermal growth factor receptor; ICI, immune checkpoint inhibitor; n, number; NSCLC, non‐small cell lung cancer; PS, performance‐status; TKI, tyrosine kinase inhibitor.

^a^ Eastern Cooperative Oncology Group performance status (ECOG PS) scores range from 0 to 4, with high numbers indicating high disability.

^b^ Results calculated with Fisher's exact test.

^c^ Results calculated with the Mann–Whitney U test.

## DISCUSSION

Platinum rechallenge with paclitaxel‐carboplatin and bevacizumab as a late‐line therapy is effective for most patients with nonsquamous NSCLC and the associated AEs are tolerable.

Asahina et al. reported that 38.4% of patients with advanced NSCLC who received first‐line chemotherapy could receive third‐line chemotherapy.^12^ Some patients treated with second‐ or later‐line therapies maintain good performance status. However, data on the efficacy and safety of third‐ or later‐line chemotherapy for advanced NSCLC are limited.^13^ Patients with NSCLC who have received several lines of therapy are less likely to be eligible for clinical trials. This is mainly because their treatment histories are diverse, which may affect the efficacy and safety of the trial. Therefore, in these cases, observational studies in actual clinics may prove worthwhile.

Data on platinum rechallenge in patients with NSCLC are limited. In a pooled analysis,^14^ the response rate to platinum rechallenge with pemetrexed or taxanes was 27% in patients with NSCLC who relapsed after first‐line platinum therapy. However, the response rates and median PFS were better with taxanes than with pemetrexed. Although the above analysis included some patients on third‐line therapy or later, most patients were on second‐line therapy. To date, no study has focused only on patients on third‐line therapy or later. Furthermore, to the best of our knowledge, there is no trial of platinum rechallenge for only patients with NSCLC who relapsed after second‐ or later‐line therapy. In recent years, the preferred first‐line therapies for nonsquamous NSCLC are platinum plus pemetrexed, platinum plus pemetrexed with bevacizumab, and platinum plus pemetrexed with pembrolizumab, and so on. On the other hand, recent findings regarding an intertwined regulation of VEGF signaling and immunosuppression in the tumor microenvironment suggest that the combination of ANTI‐VEGF agents and ICIs could have synergistic antitumor activity.^15^ After treatment with ICIs, treatment with bevacizumab might have better clinical efficacy. Furthermore, it is just a guess, but angiogenesis inhibitors such as bevacizumab might improve the efficacy of later ICI rechallenges. In the present study, we empirically analyzed paclitaxel‐carboplatin with bevacizumab as a late‐line therapy in the real‐world.

In the present study, the median OS and median PFS in patients with nonsquamous NSCLC following paclitaxel‐carboplatin with bevacizumab treatment as the late‐line therapy were 11.8 and 6.3 months, respectively. The ORR in these patients was 66.7%. The ECOG 4599 trial, a randomized phase 3 study involving patients with advanced nonsquamous NSCLC who had not previously undergone chemotherapy, compared paclitaxel and carboplatin alone with paclitaxel and carboplatin plus bevacizumab. In the paclitaxel and carboplatin plus bevacizumab group, the median survival was 12.3 months and the median PFS was 6.2 months. The ORR in these patients was 35%. Thus, the median OS and median PFS reported in our study were not considerably inferior to those in the previous study, even though our study included only patients on third‐line therapy or later. Moreover, the ORR in this study (66.7%) was higher than that reported in the previous study.

In previous large clinical randomized trials of docetaxel plus ramucirumab therapy, docetaxel monotherapy, pemetrexed monotherapy, and tegafur/gimeracil/oteracil therapy (the S‐1 regimen), the median PFS and OS of second‐ or third‐line therapy for NSCLC were 2.9–4.5 and 7.5–12.8 months, respectively.^16^, ^17^, ^18^, ^19^ In general, as the number of lines of treatment increases, the likelihood of a positive outcome decreases. However, the results of our study are comparable to those of the previous studies.

In this study, as more than 80% of patients had received docetaxel as prior therapy, it was difficult to compare the difference in response between patients with and without previous docetaxel treatment. However, the 6.3‐month PFS and 66.7% ORR are still noteworthy and this regimen is promising.

Efficacy, PFS, and OS were not significantly different between patients with and those without an EGFR mutation. Therefore, paclitaxel‐carboplatin with bevacizumab as a late‐line therapy has the same effect regardless of the presence or absence of EGFR mutations.

In the ECOG 4599 trial, 25.5% of the patients treated with paclitaxel and carboplatin plus bevacizumab experienced neutropenia of grade 4. In the present study, the frequency of neutropenia (26.7%) was almost the same. Furthermore, most of the patients (86.7%) were treated with up to four courses of therapy, suggesting that these AEs were manageable.

In the univariate analysis of this study, responders were significant younger than nonresponders (*p* = 0.012). There was no significant difference between responders and nonresponders in terms of prior treatment with ICI or EGFR TKIs. Further research is needed and there is a possibility that this treatment may be more appropriate for younger patients.

There were some limitations in the present study. First, this was a retrospective study performed in a single institution using a small number of patients. In general, a small number of patients were in good general condition after second‐ or later‐line treatments. The patients of this study had good performance status. However, they had already been heavily treated, and their prognosis was not very good, with a few treatment options left as a late‐line treatment. Second, observer bias cannot be ruled out. Third, previous medications may have altered the effectiveness and safety of this treatment. Finally, although only three patients had been treated with ICIs as a first‐line therapy, ICI were administered as a second‐ or late‐line therapy in most patients. Currently, more patients are treated with ICI monotherapy or ICI plus chemotherapy as the first‐line therapy; thus, the efficacy and safety of paclitaxel‐carboplatin with bevacizumab as a subsequent treatment to these therapies requires future consideration. As the number of treatment options has increased in recent years, the sequence of treatments and overall therapeutic strategy are becoming increasingly important. In this light, this platinum rechallenge with paclitaxel‐carboplatin and bevacizumab may be a promising treatment option.

In conclusion, platinum rechallenge with paclitaxel‐carboplatin and bevacizumab as a late‐line therapy may be an effective option in patients with nonsquamous NSCLC, regardless of the presence or absence of EGFR mutations. Moreover, the AE associated with this treatment were tolerable. Further examinations such as prospective studies comparing this regimen with docetaxel or S‐1 are warranted.

## AUTHOR CONTRIBUTIONS

Jun Sugisaka: Conceptualization, investigation, formal analysis, writing–original draft, and writing–review and editing. Yukihiro Toi: Conceptualization, investigation, formal analysis, writing–original draft, and writing–review and editing. Yosuke Kawashima: Investigation, writing–original draft, and writing–review and editing. Yutaka Domeki: Investigation, writing–original draft, and writing–review and editing. Tomoiki Aiba: Investigation, writing–original draft, and writing–review and editing. Sachiko Kawana: Investigation, writing–original draft, and writing–review and editing. Atsushi Nakamura: Investigation, writing–original draft, and writing–review and editing. Shinsuke Yamanda: Investigation, writing–original draft, and writing–review and editing. Yuichiro Kimura: Investigation, writing–original draft, and writing–review and editing. Shunichi Sugawara: Conceptualization, methodology, formal analysis, writing–original draft, writing–review and editing, and project administration.

## FUNDING INFORMATION

This research received no specific grant from any funding agency in the public, commercial, or not‐for‐profit sectors.

## CONFLICT OF INTEREST STATEMENT

Shunichi Sugawara received honoraria from MSD, Ono Pharmaceutical, Bristol‐Myers Squibb, AstraZeneca, Chugai Pharma, Nippon Boehringer Ingelheim, Pfizer, Taiho Pharmaceutical, Eli Lilly and Company, Novartis, Kyowa Kirin, and Yakult Honsha. Yukihiro Toi received honoraria from MSD, Ono Pharmaceutical, AstraZeneca, Taiho Pharmaceutical, and Bristol‐Myers Squibb. Yosuke Kawashima received honoraria from Eli Lilly and Company and Taiho Pharmaceutical. Atsushi Nakamura received honoraria from Nippon Boehringer Ingelheim, Chugai Pharma, MSD, and AstraZeneca. Shinsuke Yamanda received honoraria from AstraZeneca, Taiho Pharmaceutical, Novartis, and GlaxoSmithKline. The other authors declare no conflicts of interest.
